# Editorial: Visual mismatch negativity (vMMN): A unique tool in investigating automatic processing

**DOI:** 10.3389/fnhum.2022.1056208

**Published:** 2022-11-07

**Authors:** Piia Astikainen, Kairi Kreegipuu, István Czigler

**Affiliations:** ^1^Department of Psychology, University of Jyvaskyla, Jyväskylä, Finland; ^2^Institute of Psychology, University of Tartu, Tartu, Estonia; ^3^Institute of Cognitive Neuroscience and Psychology, Research Centre for Natural Sciences (RCNS), Budapest, Hungary

**Keywords:** visual mismatch negativity (visual MMN), event-related potentials, change detection, preattentive processing, visual system

Since 1978, when mismatch negativity (MMN) was reported for the first time in the auditory modality (Näätänen et al., 1978), MMN has been used to investigate automatic change detection. The first reports of visual mismatch negativity (vMMN) were published ~20 years after the discovery of the auditory MMN (Tales et al., 1999). Thereafter, vMMN has been used to study basic visual feature processing (Czigler et al., 2002; Astikainen et al., 2008; Grimm et al., 2009) as well as complex visual information processing, mostly face information (Astikainen and Hietanen, 2009; Kreegipuu et al., 2013), in normative and clinical populations (for a review see, Kremláček et al., 2016).

Later, vMMN, as well as its auditory counterpart, has been linked to predictive coding theory (Stefanics et al., 2014), which states that the brain predicts future events based on the representations of previous sensory events (Friston, 2005). This is well in line with the original idea of Risto Näätänen that the MMN reflects a memory-comparison process between the sensory memory of the standards and the “deviant” sensory input (Näätänen, 1990). In predictive coding framework (v)MMN is suggested to reflect prediction error which occurs when the new sensory input does not fit the predicted model (Friston, 2005). Predictive coding is suggested to be aberrant in several psychiatric conditions (for a recent review, see Smith et al., 2021). Therefore, vMMN is an important tool to investigate also predictive mechanisms in neuropsychiatric conditions, and it may have the potential for clinical applications.

The present Research Topic includes seven articles: six articles report empirical investigations of automatic visual deviance detection, while one article reviews visual mismatch negativity (vMMN) findings. Each of these articles is an important contribution to developing a better understanding of automatic visual cognition.

Four studies applied faces as stimuli to elicit the vMMN. Zeng et al. utilized an oddball and an equiprobable stimulus condition to disentangle the effects of probability and low-level features of the stimuli on vMMN. The vMMN amplitude was affected by neither of them. The results thus associate the vMMN to face stimuli to the memory-comparison theory of the mismatch negativity (Näätänen et al., 2005).

Two articles studied the possible “coarse-to-fine” principle in face processing. The article by Wang et al. investigated configural (i.e., the distance between eyes and between mouth and nose) and featural (i.e., the shape of the eye and mouth) information processing in non-attended faces. They found that the vMMN emerged in the 200–360 ms latency for the configural, but not the featural, face information. This suggests that different mechanisms underlie automatic configural and featural face processing. Lacroix et al. investigated the effects of low and high spatial frequencies in faces on vMMN. The vMMN was larger for high spatial frequency than for low spatial frequency faces approximately at 300–400 ms post-stimulus latency, reflecting lower prediction error for low than high spatial frequencies. Also, P100 was found to be more sensitive to low spatial frequencies, while the face-sensitive N170 was more sensitive to high spatial frequencies. This pattern could be in line with the coarse-to-fine hypothesis.

Ford et al. investigated vMMN to neutral, happy, and sad faces in healthy adults. They found that the vMMN amplitude to happy faces tended to correlate with some interpersonal features associated with autism and schizophrenia. Higher levels of interpersonal difficulty were found in those people who had a larger amplitude of the vMMN to happy faces. The article contributes to the growing literature searching for brain activity markers of neuropsychiatric conditions.

The article by Cheng et al. applied Chinese characters as stimuli when investigating the visual processing of self-referential information. The names of participants' birthplaces and University cities were applied as rarely presented deviant stimuli in a series of standard stimuli representing irrelevant cities. The results demonstrated a vMMN, which was strongest in the left occipital–temporal region, to the birthplace, but not to the University city. These findings suggest that visually presented birthplace information can be automatically processed similarly to own face and name information that has been previously studied in the context of self-referential information.

Kurita et al. investigated whether visual change detection can affect access processing to visual awareness (APVA). vMMN amplitude and perceptual alternation in binocular rivalry were measured to the orientation deviant that was perceived unconsciously, and the facilitation of APVA by the deviant was estimated. The relationship between vMMN and APVA was investigated by means of inter-individual variability and the correlation between vMMN enhancement and an increase in the proportion of perceptual alternation across participants. The results demonstrated that the unconsciously presented deviant stimulus made the unconscious stimulus more likely to reach consciousness. In addition, an association between the increased vMMN amplitude and the facilitation of perceptual alternation in binocular rivalry when an unconscious deviant was presented was found.

Czigler and Kojouharova reviewed 12 studies investigating vMMN in non-clinical populations (athletes, internet addicts), the effects of specific conditions (hypoxia, mental fatigue), and the sensitivity of vMMN to special stimuli, like artwork.

Altogether the Research Topic extends our understanding of preattentive visual processing, and it strongly contributes to face processing literature. The whole set of articles validates vMMN as an applicable tool to estimate the preattentive processing of visual stimuli whenever attentional processing or verbal reports are not accessible.

## Author contributions

PA drafted the manuscript. KK and IC revised the text. All authors contributed to the article and approved the submitted version.

## Funding

KK was supported by the Estonian Research Council grant (PRG1151). PA was supported by the Academy of Finland (Grant Number: 2100005537).

## Conflict of interest

The authors declare that the research was conducted in the absence of any commercial or financial relationships that could be construed as a potential conflict of interest.

## Publisher's note

All claims expressed in this article are solely those of the authors and do not necessarily represent those of their affiliated organizations, or those of the publisher, the editors and the reviewers. Any product that may be evaluated in this article, or claim that may be made by its manufacturer, is not guaranteed or endorsed by the publisher.
